# 

**Published:** 1894-04

**Authors:** 


### CONTENTS.

### COMMUNICATIONS.

Diseases of the Maxillary Bones and their Periosteum.................. 157
Trional.................................................................... 163
Enemies of the Human Teeth............................................ 164
Practical Oral Therapeutics........................................... 172
Reforms.................................................................... 187
Therapeutics of Aconite and Iodine in Pericementitis.................. 191
Infection from the Mouth.............................................. 194

### SELECTIONS.

The Mind and the Weather.............................................. 199
Hygienic................................................................... 201

### NOTICES.

The San Francisco Dental Association................................ 203
Joint Meeting of the Iowa and Nebraska State Dental Societies....... 203
Northern Ohio Dental Association...................................... 203

### CORRESPONDENCE.

For Regulating Teeth.................................................... 205
The Mississippi Valley Dental Association........................... 206

### EDITORIAL.

Bibliographical........................................................... 207

